# Exposure and Effect Assessment of Aerosolized Red Tide Toxins (Brevetoxins) and Asthma

**DOI:** 10.1289/ehp.0900673

**Published:** 2009-04-13

**Authors:** Lora E. Fleming, Judy A. Bean, Barbara Kirkpatrick, Yung Sung Cheng, Richard Pierce, Jerome Naar, Kate Nierenberg, Lorraine C. Backer, Adam Wanner, Andrew Reich, Yue Zhou, Sharon Watkins, Mike Henry, Julia Zaias, William M. Abraham, Janet Benson, Amy Cassedy, Julie Hollenbeck, Gary Kirkpatrick, Tainya Clarke, Daniel G Baden

**Affiliations:** 1 National Science Foundation National Institute of Environmental Health Sciences Oceans and Human Health Center, University of Miami Rosenstiel School of Marine and Atmospheric Sciences, Miami, Florida, USA; 2 University of Miami Miller School of Medicine, Miami, Florida, USA; 3 Children’s Hospital Medical Center and University of Cincinnati, Cincinnati, Ohio, USA; 4 Mote Marine Laboratory, Sarasota, Florida, USA; 5 Lovelace Respiratory Research Institute, Albuquerque, New Mexico, USA; 6 Center for Marine Science Research, University of North Carolina at Wilmington, Wilmington, North Carolina, USA; 7 National Center for Environmental Health, Centers for Disease Control and Prevention, Atlanta, Georgia, USA; 8 Florida Department of Health, Tallahassee, Florida, USA

**Keywords:** asthma, brevetoxins, harmful algal blooms (HABs), Karenia brevis, red tides, sensitive populations, spirometry

## Abstract

**Background:**

In previous studies we demonstrated statistically significant changes in reported symptoms for lifeguards, general beach goers, and persons with asthma, as well as statistically significant changes in pulmonary function tests (PFTs) in asthmatics, after exposure to brevetoxins in Florida red tide (*Karenia brevis* bloom) aerosols.

**Objectives:**

In this study we explored the use of different methods of intensive ambient and personal air monitoring to characterize these exposures to predict self-reported health effects in our asthmatic study population.

**Methods:**

We evaluated health effects in 87 subjects with asthma before and after 1 hr of exposure to Florida red tide aerosols and assessed for aerosolized brevetoxin exposure using personal and ambient samplers.

**Results:**

After only 1 hr of exposure to Florida red tide aerosols containing brevetoxin concentrations > 57 ng/m^3^, asthmatics had statistically significant increases in self-reported respiratory symptoms and total symptom scores. However, we did not see the expected corresponding changes in PFT results. Significant increases in self-reported symptoms were also observed for those not using asthma medication and those living ≥ 1 mile from the coast.

**Conclusions:**

These results provide additional evidence of health effects in asthmatics from ambient exposure to aerosols containing very low concentrations of brevetoxins, possibly at the lower threshold for inducing a biologic response (i.e., toxicity). Consistent with the literature describing self-reported symptoms as an accurate measure of asthmatic distress, our results suggest that self-reported symptoms are a valuable measure of the extent of health effects from exposure to aerosolized brevetoxins in asthmatic populations.

Florida red tides are a manifestation of the larger growing environmental issue of harmful algal blooms (HABs) (Erdner et al. 2008; Van Dolah 2000). HABs are blooms of micro-algae in aquatic environments, causing harm to humans and other animals, particularly by the production of potent natural toxins. Florida red tide is caused by the marine dinoflagellate *Karenia brevis*, which produces the potent neurotoxins brevetoxins. These toxins cause the death of millions of fish annually in the Gulf of Mexico, as well as morbidity and mortality among marine mammals and sea birds. Exposure to seafood contaminated by brevetoxins is associated with neurotoxic shellfish poisoning in humans, whereas the aerosols of the Florida red tide have been reported to cause respiratory irritation in persons recreating and working in coastal communities during active blooms with onshore winds (Backer et al. 2003, 2005; Fleming et al. 2005a, 2005b, 2007; Watkins et al. 2008; Zaias et al., in press).

Our prior research demonstrated that persons with a physician diagnosis of asthma experience a statistically significant change in their respiratory symptoms and pulmonary function after a 1-hr visit to the beach during a documented Florida red tide; these changes are not seen when the same individuals visit the beach for 1 hr during a non-Florida red tide period (Fleming et al. 2007; Milian et al. 2007). As part of our research, at the same time we collected symptom and pulmonary function data before and after the beach exposure for each individual subject, we collected substantial ambient samples including water for *K. brevis* cell counts and brevetoxin concentrations; ambient aerosol samples for brevetoxin concentrations and aerosol particle size; and individual personal air samples for brevetoxin concentrations (Cheng et al. 2005a, 2005b, 2005c; Naar et al. 2002; Pierce et al. 2003). In the present study we investigated for the first time which of the meas ures of brevetoxin concentrations in ambient samples were most closely associated with health effects and whether there were dose–response relationships between these ambient measurements and health outcomes.

## Methods

An interdisciplinary team of researchers from federal, state, private, and local organizations have been evaluating aerosolized *K. brevis* red tide brevetoxin exposures and their possible acute and chronic adverse health effects in humans and animals. The study has been approved by the participating institutional review boards. The study location was Siesta Beach (Sarasota, FL), where prolonged Florida red tides lasting months have become almost an annual event. Although the asthmatic cohort has been studied since 2003, the data used in this analysis were from Florida red tide exposure periods in March 2005 and September 2006.

The study participants consisted of an open cohort of asthmatics with the following characteristics: a self-report of physician-diagnosed asthma; ≥ 12 years of age, history of smoking ≤ 10 years; able to walk on the beach continuously for at least 30 min; and at least 6 months residence in the Sarasota area. For our study, participants spent ≥ 1 hr at the beach in areas with ongoing ambient monitoring. During this time, they could return from the beach at any time if they felt symptomatic, and all participants were encouraged to use any personal medications as needed before, during, and after the study period. Participants were asked not to change their daily asthma management regime on the day of the study.

After obtaining informed consent, we collected detailed baseline information for all subjects (including their medical history and possible confounders) in a baseline questionnaire. Each subject participated in at least one evaluation during an active *K. brevis* bloom (exposure period) and in one evaluation during a period when there was not a bloom (nonexposure period), although only the exposure period data were used for the analysis. Both evaluations included prebeach and postbeach questionnaires, nasal swab sampling, and spirometry. The prebeach and postbeach questionnaires collected information on recent medical history and medication use, as well as symptoms and possible confounders (e.g., smoking).

Each study participant carried an IOM personal air monitor (SKC Inc., Eighty Four, PA) during their 1 hr of beach exposure. Based on the results of ambient monitoring, the personal monitoring for brevetoxins is important, as the aerosols can vary widely within a stretch of beach, due to wind gusts and direction, the patchy nature of *K. brevis* blooms, and variation between participants depending on where and when she/he walked on the beach during an active Florida red tide bloom (Cheng et al. 2005a, 2005b, 2005c).

Pulmonary function testing was performed according to National Institute for Occupational Safety and Health (NIOSH) standards (NIOSH 1997) using a portable OMI2000 10-L dry rolling seal volume spirometer (Occupational Marketing, Inc., Houston, TX) before and after 1-hr beach exposure. The spirometry values were forced expiratory volume in 1 sec (FEV_1_); the average rate of flow during the middle half of a forced vital capacity (FVC) maneuver (FEF_25–75_); peak expiratory flow (PEF), and FVC. Only data conforming to the standard guidelines for collection and interpretation of spirometry measurements were accepted, and all study participants had ≥ 3 reproducible spirograms before and after visiting the beach (Fleming et al. 2007; NIOSH 1997).

### Ambient monitoring

For ambient monitoring, a portable, self-contained weather station near a high-volume air particle impactor and the ambient air samplers was used to monitor the air temperature, relative humidity, wind speed, and direction, as described previously (Cheng et al. 2005a, 2005b, 2005c; Naar et al. 2002; Pierce et al. 2003). Water samples were collected twice daily in 1-L glass bottles from the surf zone adjacent to the high-volume air sampler locations. The water samples were analyzed for *K. brevis* cell counts and for brevetoxin concentrations using both the brevetoxin enzyme-linked immunosorbent assay (ELISA) and liquid chromatography-mass spectroscopy (LC-MS) analysis (Fleming et al. 2007; Naar et al. 2002; Pierce et al. 2003).

Air samples for toxin and particulate size were collected using two types of high-volume samplers (high-volume filter and high-volume air particle impactors equipped to capture aerosol particles by size) and a personal sampler of the subject breathing zone. We measured brevetoxins by the high-volume sampler and the personal sampler. For the high-volume filter samplers, six 4-hr air filter samples and eight 1-hr air filter samples were measured at a sampling flow rate between 1.6 and 2.0 m^3^/min. The 1-hr personal exposure of each participant was measured using an individual personal air sampler placed near the breathing zone; the sampling flow rate for the personal samplers was 9-L/min (Cheng YS, personal communication). For the personal samples, we used only the brevetoxin ELISA because it had a lower limit of detection (LOD) than LC-MS, which is important given the low flow rate of the personal air sampler and the small size of the filter paper to be analyzed for brevetoxins. The ambient samples could be analyzed by both LC-MS and brevetoxin ELISA because the flow rate of the ambient air sampler was substantially higher and the filter paper was larger.

The brevetoxin ELISA measures any substance (parent toxins and toxins derivatives/ metabolites) containing the brevetoxin type 2 backbone structure. As such, the reported concentration represents the total amount of brevetoxin-like compounds present in the sample and may also include toxins and/or derivatives that have not yet been chemically described. The LC-MS levels reported in this study represent only the sum of the concentration of five specific breve toxins that could be present in a given sample (PbTx-1, PbTx-2, PbTx-3, PbTx-9, and PbTx-3 42-carboxylic acid). During the 2005 and 2006 exposure periods, the LOD for individual brevetoxins in seawater was 0.03 μg/L using LC-MS and 0.6 ng/sample using the brevetoxin ELISA for all brevetoxins. The LOD for the LC-MS analysis of the ambient air samples was 0.01 ng/m^3^ for individual brevetoxins; the ELISA LOD for both the ambient and personal samples was 0.6 ng/sample for all brevetoxins combined.

### Exposure and health assessment

In previous analyses involving the asthmatic cohort, “exposure” was defined as spending 1 hr on the beach during a study day when *a*) *K. brevis* cells above background levels (i.e., > 5,000 cells/L) and brevetoxins were detected by LC-MS and ELISA in the water, and *b*) brevetoxins were detected in the ambient air monitors by LC-MS and ELISA (Fleming et al. 2005b, 2007; Milian et al. 2007). However, no attempt was made previously to assign individual exposure levels or to use the personal air sampler data. In this analysis, each subject who had participated in a study during an active Florida red tide was assigned *a* ) the ELISA brevetoxin level from their individual personal air sampler (personal ELISA); *b*) the ELISA brevetoxin level from the hourly ambient air sampler (ambient ELISA) that corresponded to their individual beach walk time; and *c*) the LC-MS brevetoxin level from the hourly ambient air sampler (ambient LC-MS) that corresponded to their individual beach walk time. The ambient sampler brevetoxin concentrations were calculated from the hourly samples corresponding to the sampling time of each personal sample. When a personal sample was taken during a portion of two hourly ambient samples, the corresponding ambient concentration was calculated by multiplying the hourly sample with the fraction of time the personal sample was taken during each of the particular hourly samples and then summing the two concentrations.

We asked questions about the presence of symptoms consistent with asthma (i.e., eye, nose, and throat irritation, cough, wheeze, chest tightness, and shortness of breath) before and after spending 1 hr on the beach. If the participant reported any symptom, they were asked if they experienced it as mild, moderate, or severe. We analyzed the symptom data in two ways. First, the number of persons not reporting a symptom before going on the beach but reporting the particular symptom after exposure was counted as being symptomatic from exposure (Fleming et al. 2005b, 2007). Second, for those individuals reporting any respiratory symptom, we devised a respiratory symptom intensity score based on the sum of all respiratory symptoms with their individual symptom intensity of response [i.e., mild (1), moderate (2), and severe (3)] (Milian et al. 2007); the mean difference in the prebeach walk and postbeach walk symptom scores was then evaluated for each individual.

As with the symptoms, each participant served as their own spirometry control. We present the mean difference of the individual prebeach walk minus postbeach walk spirometry values. A positive mean difference indicated a decrease in lung function after the walk compared with before the walk. Because brevetoxins were measured ≥ 1 mile inshore from the coast during active *K. brevis* bloom aerosols when there were strong-enough onshore winds, “coastal residence” was defined as residence on a barrier island or along Sarasota Bay within 1 mile of a coast (Kirkpatrick et al. 2006). As in previous studies, the use of asthma medications within 12 hr before going to the beach was used as a surrogate for increased asthma severity (Fleming et al. 2007; Milian et al. 2007).

### Statistics

We created the study database in Microsoft ACCESS (Microsoft Corporation, Redmond, WA), with direct data entry during participant interviews. Descriptive and other statistical analyses were performed using SAS statistical software, version 9.1 (SAS Institute Inc., Cary, NC). For all subjects who participated in both March 2005 and September 2006, we used the data with the highest LC-MS ambient measure.

For the symptom data, analyses were performed using the distribution of the exposure measures by quartiles and by above/below the median of the ambient brevetoxin level. For pulmonary function data, we compared the mean differences between the different groups above and below the mean, the median, and by quartiles, depending on the particular data distribution. Statistical hypothesis testing was performed using paired *t*-test for continuous data and McNemar’s test for categorical data to compare pre beach and postbeach data (Kleinbaum et al. 1982). The McNemar’s test at 0.05 level of significance uses only the data on the diagonal of the two-by-two table, which indicates a change; thus, subjects who came to the beach reporting no symptom and left reporting a particular symptom were compared with subjects who came to the beach reporting a symptom and left reporting no particular symptom. For the symptom score, the pre beach walk value of each individual was compared with that individual’s post-beach walk value using a two-tailed paired *t*-test at the 0.05 level of significance. In addition, we performed correlation analyses between individual pulmonary function tests (PFTs) and the ambient measures, as well as multi variable regression analysis with differences between pre- and post-PFTs (as the outcome variable), ambient levels, medication use, sex, age, and geographic proximity of residence to the coast.

## Results

There were 87 asthmatic persons ≥ 12 years of age who participated in at least one exposed study period during 2005–2006, with a mean age (± SD) of 44.9 ± 19.2 (range, 12.0–79.0) (Table 1). The majority (56%) were white, non-Hispanic females with many years of asthma diagnosis.

### Ambient exposure

Although there were nine sampling periods from 2003 through 2008, which included 3 exposed days (March 2003, March 2005, September 2006) and 6 unexposed days (January 2003, May 2004, October 2004, February 2005, June 2007, September 2007, May 2008), we used the exposure days from March 2005 and September 2006 because the aerosol sampling equipment and analyses had been optimized in terms of air sampler flow rates and limits of detection for the brevetoxins (Table 2). Of note, the Florida red tide had been present in both cases in the Sarasota area for about 1–2 months before the study periods; the range of brevetoxins measured for these two study periods was only exceeded in one prior study with a peak brevetoxin level of 93 ng/m^3^ measured by HPLC (Backer et al. 2003; Fleming et al. 2007).

We found strong and statistically significant correlations among the personal ELISA, the ambient ELISA, and the ambient LC-MS. In particular, the personal ELISA and the hourly ambient ELISA had a correlation coefficient of 0.71 (*p* < 0.001), as did the personal ELISA and the hourly ambient LC-MS (*r* = 0.71; *p* < 0.001), whereas the ambient ELISA and LC-MS measurements were even more highly correlated (*r*= 0.91; *p*< 0.001).

### Ambient exposure and human health

Among the 87 people with a personal sample ELISA, the mean (± SD) level was 73.3 ± 78.9 ng/m^3^, with a range of 0–366.2 ng/m^3^ and a median of 45.7 ng/m^3^. For the ambient hourly sample ELISA, the mean level was 161.5 ± 96.3 ng/m^3^, with a range of 0–375.4 ng/m^3^ and a median of 141.0 ng/m^3^. For the ambient hourly sample LC-MS, the mean level was 53.4 ± 32.9 ng/m^3^, with a range of 0–117.5 ng/m^3^ and a median of 56.8 ng/m^3^.

Upon review, the hourly ambient LC-MS measurements had the strongest associations with the health end points (i.e., symptoms and pulmonary functions); therefore, only the associations between the hourly LC-MS ambient measurements of aerosolized brevetoxins and the health end points are summarized below.

### Report of symptoms

#### Symptoms yes/no

For the individuals having an hourly ambient LC-MS brevetoxin level above the median (i.e., 56.8 ng/m^3^), statistically significant differences for upper and lower respiratory tract symptoms (i.e., cough, wheeze, throat irritation, chest tightness, and nasal irritation) were seen when examining the change from “no symptom” before beach exposure to “yes symptom” after beach exposure (Table 3). For below the median, there was only a significant report of chest tightness.

We also observed statistically significant differences for both above and below the median brevetoxin level for those without medications within 12 hr of going to the beach (no medications). Those who lived ≥ 1 mile from the coast (far) had significant reports for all respiratory symptoms (data not shown). For those who lived close to the coast or reported using medications, only report of cough and chest tightness were significantly increased above the median.

We observed statistically significant differences in the asthmatics in the highest exposure quartile of hourly ambient LC-MS brevetoxin level compared with those in the lowest exposure quartile with respect to the seven respiratory symptoms (Table 3). Overall, those in the highest exposure quartile reported significantly more respiratory symptoms than those in the lowest exposure quartile.

#### Symptom scores

For the hourly ambient LC-MS brevetoxin level, the difference in the mean symptom scores pre- and post-exposure above the median brevetoxin level were statistically significant (Table 4). For below the median, we found no significant differences in the mean pre/post exposure respiratory symptom scores. In addition, the mean differences in the symptom scores were increased for the hourly ambient LC-MS above the median compared with those below the median (mean ± SD: 4.14 ± 3.46 vs. 0.32 ± 3.51, respectively).

We found statistically significant differences above the median brevetoxin level for the mean difference in the pre/post exposure respiratory symptom for those both with and without medications within 12 hr of going to the beach (Table 4). For below the median, there were no significant differences in the mean pre/postexposure respiratory symptom scores. The mean differences in the symptom scores were increased above the median brevetoxin level compared with those below the median (no medications: 3.89 ± 2.97 vs. 1.35 ± 3.86, respectively; with medications: 5.11 ± 5.06 vs. 0.67 ± 3.01, respectively). We also observed statistically significant differences above the median for the mean difference in the pre/ postexposure respiratory symptom scores for those with those who lived > 1 mile (far) and ≤ 1 mile (close) from the coast (Table 4). For below the median, there were no significant differences in the mean pre/post exposure respiratory symptom scores. The mean differences in the symptom scores were increased above the median brevetoxin level compared with those below the median (far: 4.44 ± 3.71 vs. 0.64 ± 3.03, respectively; close: 2.89 ± 2.32 vs. 0.06 ± 4.12, respectively).

### PFTs

None of the comparisons between the differences in the means above and below the median for the personal ELISA, or the hourly ambient ELISA and LC-MS brevetoxin levels were statistically significant (Table 5; data are shown only for the hourly ambient LC-MS). We found considerable variation in the pulmonary function data, and not all the mean differences were positive (i.e., demonstrating decreased pulmonary function after exposure compared with before). Only the personal ELISA PFT results demonstrated a consistent pattern where the above-the-median pulmonary function differences were greater than the below-the-median pulmonary function differences (data not shown).

The correlations between the pre/post-exposure pulmonary function differences for each individual pulmonary function measures with the personal ELISA and the hourly ambient ELISA and LC-MS brevetoxin levels were negative (i.e., as the measured brevetoxin level increased, the difference in individual pulmonary functions decreased); however, these results were non significant and very small, ranging from *r* = −0.04 to −0.14 (data not shown). Finally, using multiple regression with pre/postexposure FEV_1_ score as the outcome measure predicted by the model of ambient level, asthma medication use, sex, age, and proximity, we found no significant results. The very small *R^2^* (0.008) indicated that < 1% of the variation in mean pre/postexposure FEV_1_ score difference could be accounted for by these variables (data not shown).

## Discussion

In this study we examined the possible dose–response relationship between health effects (i.e., reported symptoms and PFT results) and exposure to brevetoxins measured during 1 hr of exposure to Florida red tide aerosols (i.e., by the personal ELISA and the hourly ambient ELISA and LC-MS). We found strong associations between the two ambient and one personal sampler brevetoxin measures. There was a statistically significant and positive dose–response relationship between reported asthma symptoms with all the ambient measures, particularly with the ambient LC-MS, for brevetoxin aerosols > 57 ng/m^3^ (the median brevetoxin level as measured by LC-MS from the hourly ambient sampler). We also observed an association between report of asthma symptoms for persons with less-severe asthma (i.e., not on medications) and persons who lived > 1 mile from the coast, but without a dose–response relationship, as measured by the hourly ambient LC-MS. No association was found between pulmonary function changes and the three brevetoxin measures using various methods of data analysis, nor was any dose–response relationship apparent.

The finding of a greater association between report of symptoms and the ambient LC-MS monitoring as opposed to the personal and ambient ELISA monitoring for brevetoxins is not unexpected. The ELISA measures many components of the brevetoxin structure, including a recently discovered compound, brevenal, that has been found to be a natural antagonist to brevetoxins (Abraham et al. 2005a, 2005b; Bourdelais et al. 2004; Naar et al. 2002). The LC-MS is specific for individual brevetoxins, particularly brevetoxin 2 and 3 (PbTx-2 and PbTx-3, respectively); these brevetoxins have been associated with the most significant respiratory effects in asthmatic sheep and other animal models (Abraham et al. 2005a, 2005b; Benson et al. 2005). In the future, we plan to measure brevetoxins using both the ELISA and LC-MS for the personal samplers because the personal samplers should more accurately capture the breathing zone of the individual compared with the ambient monitors.

In the present study we were able to detect significant differences and a dose–response relationship for the reported symptoms, but not for the PFTs. As pointed out by Stemple and Fuhlbrigge (2008), a major limitation in the interpretation of all asthma literature is the inconsistency in the definition of response to pulmonary function testing. They concluded that response must be defined as a combination of self-report of symptoms and objective measures (such as PFTs). To address this issue, in future studies we will include more precise measures of asthma severity as part of the pre-exposure health assessment, as well as assessment of exhaled condensates for inflammatory markers. Of note, in prior studies of very healthy lifeguards and general recreational beach goers exposed to Florida red tide aerosols, significant differences were detected only in the reported symptoms, not in the PFTs (Backer et al. 2003, 2005). Based on prior research on the particulate size of the Florida red tide aerosol, 75–85% of the aerosol remains in the nasal passages and does not reach the lung (Cheng et al. 2005a, 2005b, 2005c). Furthermore, based on data from the sheep model of asthma and Florida red tide, it appears that the 1-hr exposure on the beach to brevetoxins in aerosols of Florida red tide for the asthmatic study subjects may represent a relatively low exposure level at a value near the toxicity threshold (Abraham W, personal communication).

In the present study, the subjects ideally performed their pulmonary function testing as soon as they returned from their 1 hr of beach exposure, but it is possible that because of inadvertent delays in testing, air conditioning of the study testing vehicle, or use of asthma medication, any true changes in pulmonary function were not detected. Furthermore, it is also possible in some cases that detectable pulmonary function changes were delayed by hours or even days, and were thus not detected by immediate PFTs. Of note, these asthmatic subjects have reported symptoms lasting up to at least 5 days after from their 1 hr study exposure (Kirkpatrick et al. 2009). In addition, Kirkpatrick et al. (2006) reported increases in emergency room admissions for asthma, pneumonia, and bronchitis noted in coastal residents during active Florida red tides. Finally, it is also possible that the sample size of the asthmatic participants was not large enough to address the very large variability in the pulmonary function testing results typically seen in asthmatics.

In a previous study (Fleming et al. 2007) we noted that symptom scores were significantly higher for study participants reporting no use of asthma medications in the 12 hr prior to the study exposure (used as a surrogate measure of less-severe asthma) after the 1 hr of study exposure. This was also observed in the present analysis, but without a dose–response relationship to brevetoxin exposure measurement. Both coastal and inland residents have been shown to report an increased symptom score after 1 hr of study exposure in a previous study based on symptom score (Milian et al. 2007). In that analysis, only inland residents had a significant report of asthmatic symptoms, again without a dose–response relationship to brevetoxin exposure measurement. The lack of dose–response relationship may mean that asthmatics without medications or who have not had recent ambient red tide exposure because of living inland are more sensitive to the effects of brevetoxins than those using medications and living in coastal areas with constant ambient exposure. Data from the sheep model and Florida red tide indicate that the medications commonly used in asthma management mitigate the effects of the toxins (Abraham et al. 2005a, 2005b).

Another possible limitation to the present study is the reliance on self-reported data for the symptoms. However, despite variable reliability (Chen et al. 2006; Martinez-Moragon et al. 2006), self-report of symptoms is one of the cornerstones of asthmatic diagnosis and clinical care (Stemple and Fuhlbrigge 2008). Of note, an important strength of the present study was the comparison of each individual with themselves, decreasing the possible effects of individual confounders (such as smoking, obesity, and work environment). The study participants (and researchers) have no way of knowing the amount (or even presence) of exposure they received at the time of beach exposure or during the postexposure questionnaire administration, as the brevetoxin analytical results are not received for many days after the field study has been completed.

## Conclusions

In previous studies, we demonstrated statistically significant changes in reported symptoms for lifeguards, general beach goers, and asthmatics, as well as statistically significant changes in PFTs in asthmatics, after exposure to brevetoxins in Florida red tide aerosols. In the present study we explored the use of intensive ambient and personal air monitoring in the characterization of these exposures and their possible relationship to health effects in the asthmatic study population. We found that hourly ambient air monitoring for brevetoxins as measured by LC-MS was strongly associated with symptom report. Our results suggest that self-reported symptoms are a valuable measure of the extent of health effects from exposure to aerosolized brevetoxins in asthmatic populations. After only 1 hr of exposure to brevetoxins in Florida red tide aerosols, the asthmatics had statistically significant changes in their reported respiratory symptoms and symptom scores for brevetoxin aerosols > 57 ng/m^3^ (as measured by LC-MS). Significant increases in self-reported symptoms were also observed for those not using medications and those living ≥ 1 mile from the coast. These associations were not seen with the pulmonary function testing. This lack of association between brevetoxin exposure and PFT results may represent insensitivity or timing issues of the pulmonary function measurements and/or exposure to brevetoxins near the threshold of toxicity, both of which will be explored in the future.

## Figures and Tables

**Table 1:** Demographics of 87 study participants with physician-diagnosed asthma.

Variable	No. (%)
Age [years; mean ± SD (range)]	44.9 ± 19.2 (12.0–79.0)
Female	52 (59.8)
White	85 (97.7)
Hispanic	1 (1.2)
Years with diagnosis (mean ± SD)	16.5 ± 25.2
Currently use asthma medications^a^	68 (88.3)
Positive history of Florida red tide symptoms with exposure	77 (90.6)
Current smoker	8 (12.3)
Hospitalized ≥ 1 time in past year from respiratory causes	11 (13.1)
Used medications^a^ within 12 hr before study exposure^b^	30 (34.5)
Live ≥ 1 mile from coast^b^	55 (63.2)

a Asthma medications predominantly beta_2_ agonists.

b At time of ambient LC-MS brevetoxin measurement.

**Table 2:** Summary of ambient data during exposed study periods: *K. brevis* cell counts, total brevetoxins in water, and from high-volume and personal air samplers.

Date	Temperature (°C) (mean ± SD)	Humidity (%)(mean ± SD)	Wind speed (mph) (mean ± SD)	Wind direction index (mean ± SD)	Range of *K. brevis* in water (cells/L)^a^	Water: range of total brevetoxins (μg/L)^b^	High-volume air: total brevetoxins (ng/m^3^ ) (mean ± SD)^c^	Personal air: average TWA (ng/m^3^) (mean ± SD)^d^
2005								

11 Mar	18.8 ± 0.4	72 ± 3	6.1 ± 3.3	0.76 ± 0.18	54,000–200,000	2.25–11.95	39.0 ± 13.0	41.4 ± 24.7
12 Mar	18.7 ± 0.8	34 ± 7	6.1 ± 1.8	0.50 ± 0.40			27.8 ± 11.6	77.5 ± 49.2
13 Mar	20.0 ± 0.4	89 ± 2	9.5 ± 1.4	0.51 ± 0.10			21.2 ± 6.9	26.1 ± 19.5
14 Mar	20.6 ± 0.2	95 ± 1	7.6 ± 1.5	0.40 ± 0.09			22.3 ± 13.3	18.5 ± 14.2

2006								

22 Sep	29.5 ± 0.5	72 ± 2	9.3 ± 2.5	0.52 ± 0.35	694,000–4,280,000	16.69–69.26	46.7 ± 13.7	68.1 ± 64.2
23 Sep	33.3 ± 1.4	48 ± 7	5.5 ± 1.4	0.23 ± 0.29			58.7 ± 31.0	68.2 ± 97.1
24 Sep	32.6 ± 1.4	57 ± 6	6.3 ± 1.2	0.84 ± 0.17			74.7 ± 25.1	156.0 ± 90.4

TWA, time-weighted average.

a LOD of *K. brevis* = 1,000 cells/L.

b LOD of PbTx water = 0.03 μg/L.

c High-volume air sampler measured by LC-MS; LOD = 0.01 ng/m^3^.

d Personal air sampler measured by ELISA; LOD = 0.6 ng/sample.

**Table 3:** Comparisons between, above, and below the median, and between the highest 25th percentile and the lowest 25th percentile of the brevetoxin level as measured by the ambient LC-MS by pre/postexposure symptoms (*n* = 87).

			Ambient LC-MS brevetoxin level: median				Ambient LC-MS brevetoxin level: percentile			
	Overall (*n* = 87)		Below median		Above median		Lowest 25th percentile		Highest 25th percentile	
Symptoms	Pre/post (*n*)^a^	*p*-Value^b^	Pre/post (*n*)^a^	*p*-Value^b^	Pre/post (*n*)^a^	*p*-Value^b^	Pre/post (*n*)^a^	*p*-Value^b^	Pre/post (*n*) a	*p*-Value^b^
Cough	41	0.0001	11	0.13	30	0.0001	5	0.26	15	0.0001
Wheezing	17	0.01	2	0.26	15	0.0001	1	0.56	6	0.01
Throat irritation	34	0.0001	12	0.37	22	0.0001	4	0.41	13	0.0003
Shortness of breath	21	0.02	5	0.56	16	0.0003	3	1.00	9	0.01
Chest tightness	28	0.0001	12	0.02	16	0.0003	6	0.16	7	0.03
Nasal irritation	28	0.02	11	0.84	17	0.0002	8	0.80	8	0.02
Eye irritation	11	0.23	3	0.71	8	0.058	3	0.32	3	0.93
Headache	16	0.10	8	0.59	8	0.058	3	1.00	3	0.66
Itchy skin	7	0.37	2	0.41	5	0.03	1	0.56	2	0.16
Diarrhea	0	—	0	—	0	—	0	—	0	—
Other	6	0.16	3	0.66	3	0.08	3	0.32	2	—

a Persons who came to the beach with no symptom and left with a symptom.

b Evaluated by McNemar’s test.

**Table 4:** Pre/postexposure mean difference in respiratory symptom score above and below the median brevetoxin level measured by the hourly ambient LC-MS (*n* = 87).

	Below median		Above median	
	Pre/postexposure mean difference in symptom score ( ± SD)	*p*-Value^a^	Pre/postexposure mean difference in symptom score ( ± SD)	*p*-Value^a^
All participants	0.32 ± 3.51	0.57	4.14 ± 3.46	0.0001

Used asthma medications within 12 hr before study exposure				

Yes	0.67 ± 3.10	0.34	5.11 ± 5.06	0.02
No	1.35 ± 3.68	0.11	3.89 ± 2.97	0.0001

Distance of residence from coast				

Close (≤ 1 mile)	0.06 ± 4.12	0.95	2.89 ± 2.32	0.006
Far (> 1 mile)	0.64 ± 3.03	0.33	4.44 ± 3.71	0.0001

a Evaluated by paired *t*-test.

**Table 5:** PFT mean differences pre- and post-exposure above and below the median brevetoxin level as measured by ambient LC-MS (*n* = 87).

PFT	Exposure level	PFT difference (mL) (mean ± SD)	*p-*Value^a^
FEV1	Above median	27.3 ± 123.0	0.62
	Below median	39.8 ± 113.8	
FVC	Above median	10.0 ± 128.5	0.10
	Below median	61.4 ± 159.4	
FEF_25–75_	Above median	−22.3 ± 333.97	0.32
	Below median	40.2 ± 234.9	
PEF	Above median	224.3 ± 500.1	0.95
	Below median	217.9 ± 560.2	

a Evaluated by *t*-test of difference in mean differences.

## References

[b1-ehp-117-1095] Abraham WM, Bourdelais AJ, Ahmed A, Serebriakov I, Baden (2005a). Effects of inhaled brevetoxins in allergic airways: toxin-allergen interactions and treatment. Environ Health Perspect.

[b2-ehp-117-1095] Abraham WM, Bourdelais AJ, Sabater JR, Ahmed A, Lee TA, Serebriakov I (2005b). Airway responses to aerosolized brevetoxins in an animal model of asthma. Am J Respir Crit Care Med.

[b3-ehp-117-1095] Backer LC, Fleming LE, Rowan A, Cheng Y, Benson J, Pierce R (2003). Recreational exposure to aerosolized brevetoxins during Florida red tide events. Harmful Algae.

[b4-ehp-117-1095] Backer LC, Kirkpatrick B, Fleming LE, Cheng Y, Pierce R, Bean J (2005). Occupational exposure to aerosolized brevetoxins during Florida red tide events: impacts on a healthy worker population. Environ Health Perspect.

[b5-ehp-117-1095] Benson JM, Hahn FF, March TH, McDonald JD, Gomez AP, Sopori MJ (2005). Inhalation toxicity of brevetoxin 3 in rats exposed for twenty-two days. Environ Health Perspect.

[b6-ehp-117-1095] Bourdelais AJ, Campbell S, Jacocks H, Naar J, Wright JL, Carsi J (2004). Brevenal is a natural inhibitor of brevetoxin action in sodium channel receptor binding assays. Cell Mol Neurobiol.

[b7-ehp-117-1095] Chen E, Herman C, Rogers D, Oliver-Welker T, Strunk RC (2006). Symptom perception in childhood asthma: the role of anxiety and asthma severity. Health Psychol.

[b8-ehp-117-1095] Cheng YS, McDonald J, Kracko D, Irvin CM, Zhou Y, Pierce RH (2005a). Concentrations and particle size of airborne toxic algae (brevetoxin) derived from ocean red tide events. Environ Sci Technol.

[b9-ehp-117-1095] Cheng YS, Villareal TA, Zhou Y, Gao J, Pierce JH, Wetzel D (2005b). Characterization of red tide aerosol on the Texas coast. Harmful Algae.

[b10-ehp-117-1095] Cheng YS, Zhou Y, Irvin CM, Pierce RH, Naar J, Backer LC (2005c). Characterization of marine aerosol for assessment of human exposure to brevetoxins. Environ Health Perspect.

[b11-ehp-117-1095] Erdner DL, Dyble J, Brand LE, Parker MS, Stevens RC, Moore SK (2008). Centers for Oceans and Human Health: a unified approach to the challenge of harmful algal blooms. Environ Health.

[b12-ehp-117-1095] Fleming LE, Backer LC, Baden DG (2005a). Overview of aerosolized Florida red tide toxins: exposures and effects. Environ Health Perspect.

[b13-ehp-117-1095] Fleming LE, Kirkpatrick B, Backer LC, Bean JA, Wanner A, Dalpra D (2005b). Initial evaluation of the effects of aerosolized Florida red tide toxins (brevetoxins) in persons with asthma. Environ Health Perspect.

[b14-ehp-117-1095] Fleming LE, Kirkpatrick B, Backer LC, Bean JA, Wanner A, Reich A (2007). Aerosolized red tide toxins (brevetoxins) and asthma. Chest.

[b15-ehp-117-1095] Kirkpatrick B, Bean JA, Fleming LE, Backer LC, Akers R, Wanner A, Moestrup Ø, Doucette G, Enevoldsen H, Godhe A, Hallegraeff G, Luckas B (2009). Aerosolized red tide toxins (brevetoxins) and asthma: a 10 day follow up after 1 hour acute beach exposure. Proceedings of the 12th International Conference on Harmful Algae.

[b16-ehp-117-1095] Kirkpatrick B, Fleming LE, Backer LC, Bean JA, Tamer R, Kirkpatrick G (2006). Environmental exposures to Florida red tides: effects on emergency room respiratory diagnoses admissions. Harmful Algae.

[b17-ehp-117-1095] Kleinbaum DG, Kupper LL, Morgenstern H (1982). Epidemiologic Research.

[b18-ehp-117-1095] Martinez-Moragon E, Perpina M, Belloch A (2006). Does experience influence perception of dyspnea? [in Spanish]. Arch Bronconeumol.

[b19-ehp-117-1095] Milian A, Nierenberg K, Fleming LE, Bean JA, Wanner A, Reich A (2007). Reported respiratory symptom intensity in asthmatics during exposure to aerosolized Florida red tide toxins. J Asthma.

[b20-ehp-117-1095] Naar J, Bourdelais A, Tomas C, Kubanek J, Whitney PL, Flewelling L (2002). A competitive ELISA to detect brevetoxins from *Karenia brevis* (formerly *Gymnodinium breve*) in seawater, shellfish, and mammalian body fluid. Environ Health Perspect.

[b21-ehp-117-1095] NIOSH (1997). NIOSH Spirometry Training Guide.

[b22-ehp-117-1095] Pierce RH, Henry MS, Blum PC, Lyons J, Cheng Y, Yazzie (2003). Brevetoxin concentrations in marine aerosol: human exposure levels during a K brevis harmful algal bloom. Bull Environ Contam Toxicol.

[b23-ehp-117-1095] Stemple DA, Fuhlbrigge AL (2008). Defining the responder in asthma therapy. J Allergy Clin Immunol.

[b24-ehp-117-1095] Van Dolah FM (2000). Marine algal toxins: origins, health effects, and their increased occurrence. Environ Health Perspect.

[b25-ehp-117-1095] Watkins SM, Reich A, Fleming LE, Hammond R (2008). Neurotoxic shellfish poisoning. Mar Drugs.

[b26-ehp-117-1095] Zaias J, Backer LC, Fleming LE, Rabinowitz P, Conti L Harmful algal blooms (HABs). Human-Animal Medicine: A Clinical Guide to Toxins, Zoonoses, and Other Shared Health Risks.

